# Long-term outcomes in Rheumatoid Arthritis: Review of data from the ‘Basildon Inflammatory Arthritis Cohort’

**DOI:** 10.1093/rap/rkac075

**Published:** 2022-09-05

**Authors:** Kanishk Jain, Deena Laila, Anupama Nandagudi, Anurag Bharadwaj

**Affiliations:** Department of Rheumatology, Mid & South Essex Foundation Trust, Basildon University Hospital, Basildon, UK; Department of Rheumatology, Mid & South Essex Foundation Trust, Basildon University Hospital, Basildon, UK; Department of Rheumatology, Mid & South Essex Foundation Trust, Basildon University Hospital, Basildon, UK; Department of Rheumatology, Mid & South Essex Foundation Trust, Basildon University Hospital, Basildon, UK

**Keywords:** RA, cohort, epidemiology, outcomes, mortality, morbidity

## Abstract

**Objective:**

The aim was to determine outcomes in RA with long-term analysis of a real-world inception cohort.

**Methods:**

We carried out a retrospective cohort analysis of 184 patients with a new diagnosis of RA (ACR/EULAR 2010 criteria) between 2009 and 2013. Measured parameters included patient demographics, serological markers, disease activity (DAS28-CRP), treatment regimen, development of new co-morbidities and all-cause mortality.

**Results:**

Complete data were available for analysis in 171 patients, 60 men and 111 women, with a median age of 57 years and median follow-up time of 7.5 years. DAS-28 remission was achieved in 73%, with the majority continuing to require pharmacological therapy. Drug-free remission was achieved in 11.7%, whereas 3.5% remained refractory to treatment. Analysis of new co-morbidities revealed malignancy in 12.9% (*n* = 22), with lung cancer having the highest incidence (*n* = 9). Cardiovascular, pulmonary and cerebrovascular disease developed in 11.1% (*n* = 19), 5.8% (*n* = 10) and 5.3% (*n* = 9), respectively. The crude mortality rate was 19.3% (33 of 171), incidence mortality rate 174 per 10 000 person-years of follow-up and standardized mortality ratio 1.57 (95% CI 1.10, 2.17). More deaths were recorded from underlying malignancy [7.6% (*n* = 13)] than with cardiovascular disease [4.7% (*n* = 8)]. The majority of deaths occurred ≥5 years after initial diagnosis (67%).

**Conclusion:**

Long-term analysis reveals that mortality in RA remains significantly elevated compared with the general population. Additionally, this real-world study underlines malignancy as the predominant cause of morbidity and mortality in RA.

Key messages

- Mortality in the RA population remains elevated in comparison to the general population.
- Excess all-cause mortality in RA is particularly evident with long periods of follow-up, >5 years.
- Malignancy is emerging at the predominant cause of mortality and morbidity in RA.

## Introduction

RA is a chronic inflammatory disorder with manifestations extending far beyond effects on peripheral joints. It is known to promote hypertension [1, 2], atherosclerosis [3], dyslipidaemia [4] and immune dysregulation [5] and to increase the risk of developing major co-morbidities; these include ischaemic heart disease, heart failure, diabetes, cerebrovascular disease, interstitial lung disease and malignancy [6–8]. Past literature has highlighted excess all-cause mortality of ∼50% [9–12].

In the last two decades, we have witnessed a revolution in RA treatment, with focus on achieving early suppression within the immunological window of opportunity, a ‘treat-to-target’ approach and development of newer biological agents. Higher disease activity has been shown to increase development of new co-morbidities in the early stages of RA [8]. In particular, chronic inflammation has been linked to atherosclerosis [3], and its suppression might reduce cardiovascular risk. A recent cohort study has shown a decreasing trend of cardiovascular disease incidence in RA [13]. Furthermore, there is evidence of a steadily closing mortality gap [10, 14, 15], with two recent large studies noting similar levels of all-cause mortality between RA patients and the general population [16, 17].

The aim of this study was to assess whether improving outcomes in RA are reflected within the real-world setting. We intended to characterize such outcomes by ascertaining rates of disease remission, treatment continuance, refractory disease activity, development of new co-morbidity and mortality.

## Methods

### Study design

This is a retrospective study analysing data from an inception arthritis database at a district general hospital. This is maintained as electronic patient data via Microsoft Excel. The Basildon inflammatory arthritis cohort is composed of all patients newly diagnosed with RA under ACR/EULAR 2010 criteria between January 2009 and March 2013. Anonymized data of all 184 patients enrolled in this database were analysed for the present study. The following data were gathered: patient demographics, DAS-28 at baseline and last follow-up, serological markers, smoking status, treatment regimens, development of new co-morbidity and mortality. All patients were treated in line with the treat-to-target approach. The refractory rate was defined according to the EULAR definition for difficult-to-treat RA [18]. Drug-free remission was defined as a minimum of 1 year DMARD-free status and absence of joint swelling [19]. This study received approval from the Health Research Authority and the Health and Care Research Wales.

### Statistical methods

For treatment outcomes such as drug-free remission, biologic use and co-morbidities, we used a simple descriptive percentage. Person-years of follow-up were estimated from the initial date of RA diagnosis to the time of death or end of follow-up (February 2021). To allow for age- and sex-adjusted comparisons with the general population, we calculated the standardized mortality ratio (SMR). UK mortality data [20] were considered as the reference population, and indirect standardization was applied to calculate the expected number of deaths. During the period of study between 2009 and 2019, the yearly mortality rates in the UK have remained largely static [21]. Subsequently, the reference data were extrapolated to cover the median study period.

## Results

### Patient population

The baseline cohort consisted of 184 patients. Nine were excluded owing to a short follow-up period of <24 months (either lost to follow-up or transferred care), and four had a subsequent change in diagnosis. We present long-term outcomes from 171 patients, 60 men and 111 women, with a median age of 57 years (interquartile range 47–67 years) and a median follow-up time of 90 months (interquartile range 63–108 months). From the data available, 49% of the cohort were current or ex-smokers. RF was available in all patients, of which 71% were positive (*n* = 122). Anti-CCP antibodies were measured in 110 patients, of which 64% were positive (*n* = 70). A total of 39% of patients were found to be positive for both parameters (*n* = 67).

### Treatment outcomes

**Figure 1.**
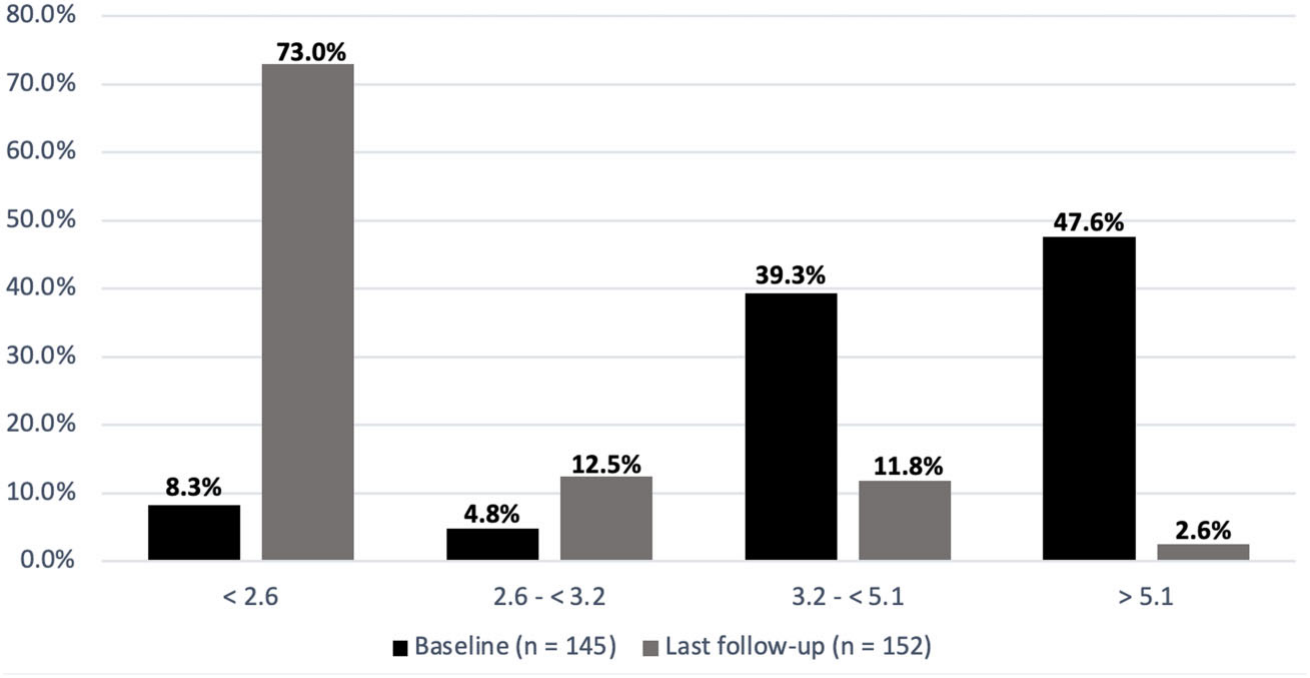
Proportion of patients with DAS-28 CRP measurement taken at baseline visit (black) and at last follow-up (grey) < 2.6: remission/no disease activity; 2.6 to < 3.2: mild; 3.2 to < 5.1: moderate; > 5.1: severe

Initial therapy at disease onset included the use of oral glucocorticoid in 32% (*n* = 55), intramuscular depomedrone in 39% (*n* = 67) and intra-articular depomedrone injection in 12% (*n* = 23). DMARDs were initiated as MTX in 63% (*n* = 108), HCQ in 20% (*n* = 35) and SSZ in 11% (*n* = 19). DAS28-CRP was available for 145 patients at baseline and 152 patients at last follow-up. Almost half of patients (*n* = 69) displayed severe disease activity at baseline; this dropped to 2.6% (*n* = 4) by the time of last follow-up (Fig. 1). At the end of the study period, 73% (*n* = 111) of patients had achieved disease remission (DAS28-CRP < 2.6), with the majority continuing to require pharmacological therapy, as single conventional synthetic DMARD (39.5%), combination conventional synthetic DMARD (29.3%) or biologic therapy (18.1%). Of the patients requiring biologic therapy, 87% (*n* = 27) were initiated on anti-TNF therapy and 13% (*n* = 3) on the anti-CD20 monoclonal antibody rituximab. A biological class switch was required in 45% (*n* = 14) of patients. Sustained drug-free remission was achieved in 11.7% of patients. A total of 3.5% of patients failed to achieve remission/low disease activity despite use of combination DMARD and at least one biologic therapy.

### Morbidity

**Figure 2.**
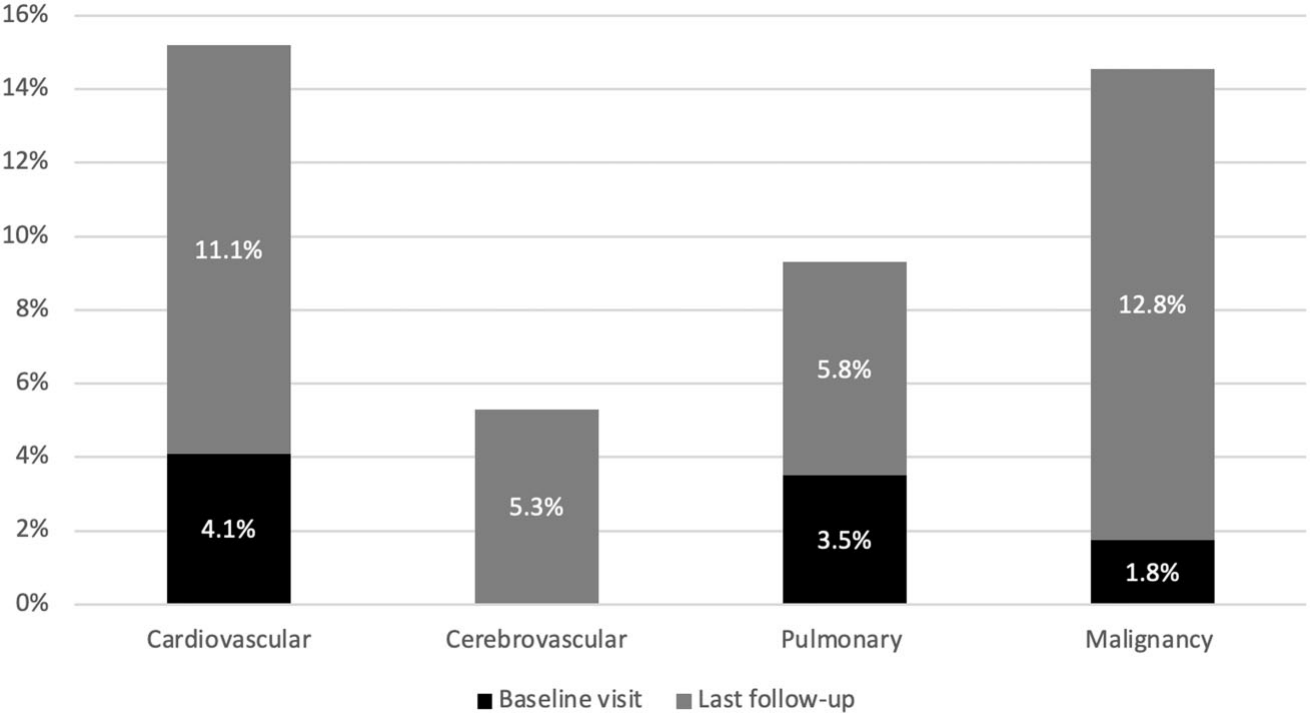
Proportion of patients with co-morbidities at baseline (black) and a new co-morbidity (grey) by the time of last follow-up

Major co-morbidities with known association to RA (ischaemic heart disease, heart failure, cerebrovascular disease, chronic obstructive pulmonary disease, interstitial lung disease, pulmonary embolism and malignancy) were physician documented at both baseline and last follow-up (Fig. 2). At baseline, seven cases of cardiovascular disease were identified, all of which were ischaemic in origin. Pulmonary disease in the form of COPD was noted in five cases and non-specific interstitial pneumonia in one case. Malignancy contributed three cases (melanoma, breast cancer and lung cancer). These cases of malignancy were of historical onset, and not within 2 years preceding RA diagnosis. No cases of cerebrovascular events were present at baseline. Cases of new co-morbidity are displayed in Table 1. No case of eye involvement, peripheral neuropathy, vasculitis, Felty’s syndrome or amyloidosis was identified. Hip replacement was required in nine patients (5.3%), knee replacement in five patients (2.9%) and corrective hand surgery in two patients (1.2%).

**Table 1.** Incidence of evaluated co-morbidities (malignancy, cardiovascular, cerebrovascular and pulmonary disease) at the time of last follow-up

Malignancy, 12.8% (22/171)	Cardiovascular, 11.1% (19/171)
Lung (9)	Ischaemic heart disease (14)
Prostate (4)	Heart failure (4)
Lymphoma (3)	Dilated cardiomyopathy (1)
Breast (2)	
Melanoma (1)	
Bladder (1)	
Pancreatic (1)	
Ovarian (1)	

**Cerebrovascular 5.3% (9/171)**	**Pulmonary 5.8% (10/171)**

Stroke (8)	Interstitial lung disease (4)
Transient ischaemic attack (1)	Pulmonary embolism (3)
	Chronic obstructive pulmonary disease (3)

### Mortality

A total of 33 deaths were noted over the study period. This constituted lung cancer (*n* = 8), heart failure (*n* = 6), dementia/frailty (*n* = 3), chronic obstructive pulmonary disease (*n* = 3), ischaemic heart disease (*n* = 2), interstitial lung disease (*n* = 2), pneumonia (*n* = 2), breast cancer (*n* = 2), ovarian cancer (*n* = 1), bladder cancer (*n* = 1), pancreatic cancer (*n* = 1), stroke (*n* = 1) and chronic liver disease (*n* = 1). The crude mortality rate was 19.3% (33 of 171). With 1205 patient-years of follow-up, the incidence mortality rate was 274 deaths per 10 000 person-years of follow-up. The age- and sex-adjusted SMR was 1.57 (95% CI 1.10, 2.17), indicating 57% excess mortality risk compared with the UK general population. On subset analysis, the mortality group displayed statistically higher (*P* < 0.017) DAS-28 scores at last follow-up (mean 2.8), compared with the alive group (mean 2.3). In contrast, baseline DAS-28 score, sex and smoking were not statistically linked to higher mortality risk.

## Discussion

Over the course of 7.5 median years of follow-up, we have tracked several parameters and outcomes for analysis. This includes cohort characteristics, disease activity, drug-free remission rate, biologic use, new co-morbidity and mortality rates.

A significant portion of the cohort reported current or prior smoking (49%); similar rates have been reported in several studies [14, 22, 23]. We obtained a DAS28-CRP remission rate of 73%, which is higher than other long-term studies: 53.5% at 5 years [24], 57.7% at 7 years [25] or 53% at 10 years [26]. DAS-28 remission rates can show variability between studies owing to differences in cohort characteristics, treatments and follow-up duration. Also, the above comparative studies displayed higher attrition rates, which could underestimate the true remission rate. The drug-free remission rate in this cohort was measured as 11.7%. This is in line with several other early arthritis cohorts: 15% in the Leiden Early Arthritis Clinic cohort [19], 9.4% in the British Early Rheumatoid Arthritis Study [19] and 15% in the BeSt Dutch study [27]. With current advances in disease suppression and monitoring, one would expect the need for biological therapy to decrease; alternatively, the cost-effective availability and use of biosimilars might increase its use. In our cohort, 18% of patients continued to require biological therapy at the end of the follow-up period. We were unable to identify measurement of this parameter in other studies, hence we could not make comparisons.

Despite all advances, there is a segment of RA that remains refractory to treatment. Based on the EULAR definition for difficult-to-treat RA, we identified 2.3% of patients with refractory disease. A community hospital cohort in Vienna identified 5.6% cases based on different criteria: use of combination DMARD and at least one biological therapy for >18 months [28]. Using this definition, our refractory rate is 3.5%. It is unclear whether different pathogenic immunological mechanisms are prevalent in refractory patients, and further research is required to identify clinical predictors.

We focused our analysis to identify development of major co-morbidities with known association to RA. Major co-morbidities in the cohort were malignancy and cardiovascular diseases. Malignancy was found to have the highest disease incidence. In particular, nine cases of lung cancer and three cases of lymphoma were documented. The association of malignancy with RA is an ongoing area of research. A recent meta-analysis showed only a modest 10% increase in risk of overall malignancy in RA compared with the general population. However, significant variation in incidence of individual malignancy types was noted, with doubled risk of malignant lymphoma and 63% excess risk of lung cancer [29]. Studies analysing the crude incidence of malignancies (excluding non-melanoma skin cancers) among four different RA registries [Consortium of Rheumatology Researchers of North America (CORRONA), Swedish Rheumatology Quality of Care Register (SRR), Norfolk Arthritis Register (NOAR) and Institute of Rheumatology, RA (IORRA)] have revealed incidence rates ranging from 72 to 136 per 10 000 person-years [30]. In comparison, the crude incidence rate in the present study is higher at 183 per 10 000 person-years.

The crude mortality rate of 19.3% in our cohort is contrasted with lower figures reported across other UK-based studies: 13% in Norfolk Arthritis Register [31], 11.6% in Clinical Practice Research Datalink [10] and 5.6% in Health Improvement Network [14]. The incidence mortality rate and SMR in the present study are relatively higher than in other RA mortality studies, which indicate an SMR of 1.15–1.29 (15–29% excess mortality risk) (Table 2). Comparisons of SMR between individual studies must be made with caution, because mortality figures can show discordance based on dissimilar variables. In particular, individual studies tend to vary in their baseline cohort characteristics (age, sex, smoking rate and baseline co-morbidity), geographical locations, time of study and duration of follow-up. The present study has a relatively small sample size, resulting in a large confidence interval. Despite these limitations, data from the present study suggest that mortality in RA continues to be high in comparison to the general population. Even at the lower end of the estimate, an excess mortality risk is observed. The mortality group had statistically higher DAS-28 scores at their last follow-up compared with the survival group; importantly, this difference was not present at baseline. This could, potentially, outline the role of unsuppressed inflammation in promoting adverse outcomes and mortality.

**Table 2.** Comparison of mortality data in RA between various UK studies and registry data

Study	Location	Incident period	Sample size	Median age (years)	Median follow-up (years)	IMR (PY)	SMR
BIAC	UK (Basildon)	2009–2013	171	57	7.5	274/10 000	1.57 (95% CI 1.10, 2.17)
NOAR Cohort 3 [31]	UK (Norfolk)	2000–2004	339	59	7	199/10 000	1.19 (95% CI 0.89, 1.60)
CPRD [10]	UK	2007–2009	21 622	61	4.3	∼200/10 000	1.15 (95% CI 1.03, 1.29)
HIN [14]	UK	2007–2014	10 769	59	3.3	170/10 000	1.29 (95% CI 1.17, 1.42)

BIAC: Basildon Inflammatory Arthritis Cohort; CPRD: Clinical Practice Research Database; HIN: Health Improvement Network; IMR: incidence mortality rate; NOAR: Norfolk Arthritis Register; PY: per 10 000 patient years of follow-up; SMR: standardized mortality ratio of all-cause mortality with 95% CI.

Cardiovascular disease is considered as the major contributor to RA mortality and accounts for approximately a third to half of premature deaths [32]. RA patients have a 1.5- to 2-fold increased risk of coronary artery disease [33] and twice the risk of developing heart failure compared with the general population [17]. However, a decreasing trend in overall cardiovascular system mortality has been reported [15, 34]. A decline in smoking rates, improvements in risk factor control, statin use and advances in interventional therapies have caused a decreasing trend in overall cardiovascular mortality [35]. A large UK study has highlighted a decline in cardiovascular system mortality in RA, but this is not reflected in neoplastic or respiratory-related deaths [10]. In the present study, neoplasms contributed to higher mortality and disease incidence than cardiovascular disease. This study suggests the emergence of malignancy as a significant driver of RA-associated morbidity and mortality.

Several factors might have contributed to the detection of poorer outcomes in this study. The follow-up time for individual patients reached a median of 7.5 years, a relatively long period in comparison to similar literature (Table 2). Most of the patients who died did so after ≥5 years of follow-up, with a median time of death at 5.5 years. There is developing consensus that several adverse outcomes in RA manifest with longer periods of follow-up [36]. Also, delayed presentation might be responsible, in part, because 70% of our patients attended the initial baseline clinic >3 months after disease onset. This might delay initiation of treatment and might impact longer-term outcomes. As previously shown, delay of DMARD initiation tends to worsen the disease course [37]. We note that our cohort had higher baseline DAS28 score in comparison to the NOAR subset (4.92 *vs* 4.31) [31], highlighting the relatively high disease activity seen in our arthritis clinic. Lastly, we also observe that the local Basildon & Thurrock region where this study was conducted has one of the highest rates of smoking and cancer-related mortality within the East of England region [38].

There are potential strengths and limitations to consider during interpretation of results. Retrospective cohort analysis introduces a risk of recall and selection bias, with access gained from the clinical database. In view of the relatively small sample size, mortality and morbidity calculations are confined within a large confidence interval. This can affect external validity and comparability to other similar studies. Furthermore, we do not have a control cohort to make direct comparisons. The major strength of our study is that we provide analysis in the real-world setting of an inception arthritis cohort. We have captured data over a relatively long follow-up period and reported low attrition rates in the process.

### Conclusion

This study shows the achievement of a high remission rate in an RA cohort with early use of combination DMARD and biologics. A segment can achieve drug-free remission, but a subset remains refractory to all treatments. All-cause mortality in RA continues to be elevated compared with the general population, with malignancy contributing higher disease incidence and mortality than cardiovascular disease.

## Data Availability

Data used in this study are stored by the corresponding author and available on request.
